# Updating the study protocol: Insight 46 – a longitudinal neuroscience sub-study of the MRC National Survey of Health and Development – phases 2 and 3

**DOI:** 10.1186/s12883-023-03465-3

**Published:** 2024-01-23

**Authors:** Heidi Murray-Smith, Suzie Barker, Frederik Barkhof, Josephine Barnes, Thomas M. Brown, Gabriella Captur, Molly R.E.Cartlidge, David M. Cash, William Coath, Daniel Davis, John C. Dickson, James Groves, Alun D. Hughes, Sarah-Naomi James, Ashvini Keshavan, Sarah E. Keuss, Josh King-Robson, Kirsty Lu, Ian B. Malone, Jennifer M. Nicholas, Alicja Rapala, Catherine J. Scott, Rebecca Street, Carole H. Sudre, David L. Thomas, Andrew Wong, Selina Wray, Henrik Zetterberg, Nishi Chaturvedi, Nick C. Fox, Sebastian J. Crutch, Marcus Richards, Jonathan M. Schott

**Affiliations:** 1 Dementia Research Centre, Queen Square Institute of Neurology, University College London, 1St Floor, 8-11 Queen Square, London, UK; 2 Department of Radiology and Nuclear Medicine, Amsterdam University Medical Center, Amsterdam, The Netherlands; 3 Centre for Medical Image Computing, University College London, London, UK; 4 Department of Neurodegenerative Disease, UCL Queen Square Institute of Neurology, London, UK; 5 MRC Unit for Lifelong Health and Ageing at UCL, Department of Population Science & Experimental Medicine, UCL Institute of Cardiovascular Science, University College London, London, UK; 6 Institute of Nuclear Medicine, University College London Hospitals, London, UK; 7 Department of Medical Statistics, London School of Hygiene and Tropical Medicine, London, UK; 8 Neuroradiological Academic Unit, UCL Queen Square Institute of Neurology, University College London, London, UK; 9 UK Dementia Research Institute, University College London, London, UK; 10 Department of Psychiatry and Neurochemistry, Institute of Neuroscience and Physiology, Sahlgrenska Academy at University of Gothenburg, Mölndal, Sweden; 11 Clinical Neurochemistry Laboratory, Sahlgrenska University Hospital, Mölndal, Sweden; 12 Hong, Kong Center for Neurodegenerative Diseases, Clear Water Bay, Hong Kong, China; 13 Wisconsin Alzheimer’s Disease Research Center, University of Wisconsin School of Medicine and Public Health, University of Wisconsin-Madison, Madison, WI USA

**Keywords:** Epidemiology, Life course, Genetics, Alzheimer’s disease, Ageing, Magnetic resonance imaging, Positron emission tomography, Cognition, Vascular disease, Birth cohort

## Abstract

**Background:**

Although age is the biggest known risk factor for dementia, there remains uncertainty about other factors over the life course that contribute to a person’s risk for cognitive decline later in life. Furthermore, the pathological processes leading to dementia are not fully understood. The main goals of Insight 46—a multi-phase longitudinal observational study—are to collect detailed cognitive, neurological, physical, cardiovascular, and sensory data; to combine those data with genetic and life-course information collected from the MRC National Survey of Health and Development (NSHD; 1946 British birth cohort); and thereby contribute to a better understanding of healthy ageing and dementia.

**Methods/Design:**

Phase 1 of Insight 46 (2015–2018) involved the recruitment of 502 members of the NSHD (median age = 70.7 years; 49% female) and has been described in detail by Lane and Parker et al*.* 2017. The present paper describes phase 2 (2018–2021) and phase 3 (2021–ongoing). Of the 502 phase 1 study members who were invited to a phase 2 research visit, 413 were willing to return for a clinic visit in London and 29 participated in a remote research assessment due to COVID-19 restrictions. Phase 3 aims to recruit 250 study members who previously participated in both phases 1 and 2 of Insight 46 (providing a third data time point) and 500 additional members of the NSHD who have not previously participated in Insight 46.

**Discussion:**

The NSHD is the oldest and longest continuously running British birth cohort. Members of the NSHD are now at a critical point in their lives for us to investigate successful ageing and key age-related brain morbidities. Data collected from Insight 46 have the potential to greatly contribute to and impact the field of healthy ageing and dementia by combining unique life course data with longitudinal multiparametric clinical, imaging, and biomarker measurements. Further protocol enhancements are planned, including in-home sleep measurements and the engagement of participants through remote online cognitive testing. Data collected are and will continue to be made available to the scientific community.

## Background

The global burden of dementia will only continue to increase as people live longer [1–3]. In 2022, the category of dementia and Alzheimer’s disease (AD) was the leading cause of deaths registered in England and Wales, accounting for 11.4% of all deaths [4]. It has been estimated that more than 850,000 people in the UK are now living with dementia, and one in 14 people aged 65 and over have dementia [5]. The economic cost of caring for people with dementia in the UK in 2019 was £34 billion per year, divided between health care (14%), social care (45%), and unpaid care (40%) [6], and these trends are representative of the global financial burden [7].

The biggest known risk factor for dementia is age, and AD and cerebrovascular disease are the most common causes of dementia and cognitive decline [8, 9]. The pathological cascades leading to dementia are not fully understood. Brain imaging biomarkers—including magnetic resonance imaging (MRI) and positron emission tomography (PET)—allow for biological processes to be investigated and quantified in vivo at an early preclinical stage [10]. In the case of AD, the deposition of ß-amyloid (Aβ) in the brain is a key and early event and likely initiates a pathological cascade that includes tau phosphorylation; dendritic, synaptic, and neuronal cell loss; inflammation; neurotransmitter failure; and cognitive decline [8]. Those processes are thought to begin 15 to 20 years before cognitive decline is observed, although it is still unknown: (a) which factors influence Aβ deposition, a critical early pathogenic step; (b) whether individuals accumulating Aβ will inevitably develop cognitive symptoms of AD dementia should they live long enough and, if so, what influences the timing of this; and (c) how Aβ interacts with other brain pathologies, including tau deposition and vascular pathology, to cause cognitive decline.

To address some of the questions and uncertainties described above, we capitalised on the unique life course data of the MRC National Survey of Health and Development (NSHD; the British 1946 birth bohort) [11], a representative, intensively studied UK birth cohort.

### MRC National Survey of Health and Development

The MRC NSHD is a nationally representative sample originally consisting of 5,362 singleton births in England, Scotland and Wales in one week of March 1946 [11]. Data have been prospectively collected through 27 waves, as well as smaller sub-study collections. Approximately 2,700 participants were in active follow-up as of 2015; of these, 2,462 completed a postal questionnaire and 1,690 attended clinic visits at ages 60–64 years. This cohort is unique in having cognitive and physical phenotypes—which cannot be accurately estimated in retrospect—measured repeatedly from childhood. Cardiovascular measures are available from age 36, blood-based biomarkers from age 53, and detailed cardiac and vascular imaging from age 60–64. Participants have demonstrated a sustained commitment to research over seven decades [12].

### Insight 46

Insight 46 is a prospective longitudinal observational neuroimaging sub-study of the NSHD. The study comprises three phases. The details of phase 1 (May 2015–January 2018) [10] and the cardiovascular measurements administered during phase 2 (January 2018–January 2021) [13] have been described previously. The present paper describes non-cardiovascular measurements administered during phase 2 and describes updates to the study protocol for phase 3 (August 2021–approximately 2026). The aims of Insight 46 are to (1) identify presymptomatic AD and associated biomarkers; (2) investigate life course and genetic influences on symptom onset and progression; and (3) provide a critical evidence base for future research.

## Methods/design

### Study organisation/funding

Phases 1 and 2 of Insight 46 were primarily funded by grants from Alzheimer’s Research UK (ARUK-PG2014-1946, ARUK-PG2017-1946 PIs Schott, Fox, Richards), the Medical Research Council Dementias Platform UK (CSUB19166 PIs Schott, Fox, Richards), and The Wolfson Foundation (PR/ylr/18575 PIs Fox, Schott; Clinical Research Fellowship, Keshavan). Phase 3 is principally funded by the Alzheimer’s Association (SG-666374-UK BIRTH COHORT PI Schott). Additional funding for Insight 46 data collection and analyses has been awarded by The Medical Research Council (MC_UU_00019/1 PI Chaturvedi and MC_UU_00019/3 PI Richards), the Wellcome Trust (Clinical Research Fellowship 200,109/Z/15/Z, Parker), Guarantors of Brain (Brain ‘Entry’ Clinical Fellowship, King-Robson), The Galen and Hilary Weston Foundation (UB170045 PI Schott), Brain Research UK (UCC14191 PI Schott), the British Heart Foundation (PG/17/90/33415 PI Hughes), and the National Brain Appeal. AVID Radiopharmaceuticals (a wholly owned subsidiary of Eli Lilly) provided the [18F]florbetapir Aβ PET tracer in kind during phases 1 and 2. Life Molecular Imaging, part of Life Healthcare’s Alliance Medical, provides the [18F]florbetaben Aβ PET tracer in kind during phase 3. Neither AVID Radiopharmaceuticals nor Life Molecular Imaging had any part in the design of the study.

As described previously [10], the sub-study was approved by the Health Research Authority Research Ethics Committee (HRA REC) London (REC reference 14/LO/1173, PI Schott). Subsequently, we have received approval for three substantial and ten non-substantial amendments that reflect the changes described in the present document. All participants provide written informed consent to participate at the beginning of each of the three phases, in accordance with the Declaration of Helsinki, and for their data to be stored in accordance with the Data Protection Act (2018) and General Data Protection Regulations (GDPR).

## Participant recruitment

### Participants

A sample of 502 NSHD study members were recruited for and consented to participate in phase 1 [10, 14], and all 502 study members were invited to participate in phase 2. Table 1 shows the numbers and characteristics of participants who a) took part in phases 1 and 2, and b) consented to phase 3 as of June 2023.

**Table 1:** Participant characteristics during Insight 46 phase 1 (complete), phase 2 (complete), and phase 3 (underway at the time of publication, numbers in table are accurate as of June 2023)

		**Phase 1**		**Phase 2**		**Phase 3 (as of June 2023)**	
**Clinic visit in London**		**n (%)**	**Median age in yrs (min–max)**	**n (%)**	**Median age in yrs (min–max)**	**n (%)**	**Median age in yrs (min–max)**
	Male	256 (51.0)	70.7 (69.2–71.7)	217 (52.5)	72.9 (71.9–74.7)	131 (52.4)	76.5 (75.5–77.3)
	Female	246 (49.0)	70.7 (69.3–71.8)	196 (47.5)	73.0 (72.0–74.1)	119 (47.6)	76.4 (75.5–77.3)
	Total	502 (100.0)	70.7 (69.2–71.8)	413 (100.0)^a^	73.0 (71.9–74.7)	250 completed, target *n* = 750	76.4 (75.5–77.3)
**Aβ** **PET/MRI** **scan**		**n (%)**	**Median age in yrs (min–max)**	**n (%)**	**Median age in yrs (min–max)**	**n (%)**	**Median age in yrs (min**–**max)**
	Male	241 (51.2)	70.7 (69.2–71.8)	191 (51.8)	72.9 (71.9–74.7)	53 (47.7)	76.3 (75.6–77.3)
	Female	230 (48.8)	70.7 (69.3–71.9)	178 (48.2)	73.0 (72.0–74.6)	58 (52.3)	76.3 (75.6–77.3)
	Total	471 (100.0)	70.7 (69.2–71.9)	369 (100.0)	73.0 (71.9–74.7)	111^b^ completed, target *n* = 500	76.3 (75.6–77.3)
**Tau** **PET/MRI scan**		n/a		n/a		**n (%)**	**Median age in yrs (min**–**max)**
	Male					63 (57.8)	76.5 (75.6–77.3)
	Female					46 (42.2)	76.7 (75.6–77.3)
	Total					109 completed, target *n* = 250	76.6 (75.6–77.3)
**Lumbar punctures**		n/a		**n (%)**		**n (%)**	
	Male			85 (63.4)		70 (61.4)	
	Female			49 (36.6)		44 (38.6)	
	Total			134 (100.0)^c^		114 (100.0)^d^	
**Remote research assessments via computer**		n/a		**n (%)**		n/a	
	Male			11 (55.0)			
	Female			9 (45.0)			
	Total			20 (100.0)			
**Remote research assessments via telephone**		n/a		**n (%)**		n/a	
	Male			5 (55.6)			
	Female			4 (44.4)			
	Total			9 (100.0)			

^a^Two participants are included in the clinic visit category who completed some assessments remotely via video conferencing as COVID-19 restrictions began to ease

^b^9 PET/MRI scans in phase 3 currently due to be rescheduled as a result of tracer production failures

^c^157 lumbar punctures were attempted in phase 2 (complete), and 134 of those procedures resulted in successful collection of cerebrospinal fluid

^d^120 lumbar punctures have been attempted in phase 3 as of June 2023 (ongoing), and 114 of those procedures resulted in successful collection of cerebrospinal fluid. Seventy-three participants have serial lumbar puncture collections to date from phases 2 (complete) and 3 (ongoing)

Four hundred thirteen participants attended a clinic visit in London for both phases 1 and 2. Of those, 367 participants had PET and MRI data acquired at both time points, with a median of 2.4 years between the two scans (minimum = 2.0 years, maximum = 3.5 years). Reasons for not completing the PET/MRI scan at one or both time points included claustrophobia, scheduling issues, implants not compatible with the MRI, and COVID-19 lockdowns (phase 2 only).

**Fig. 1:**
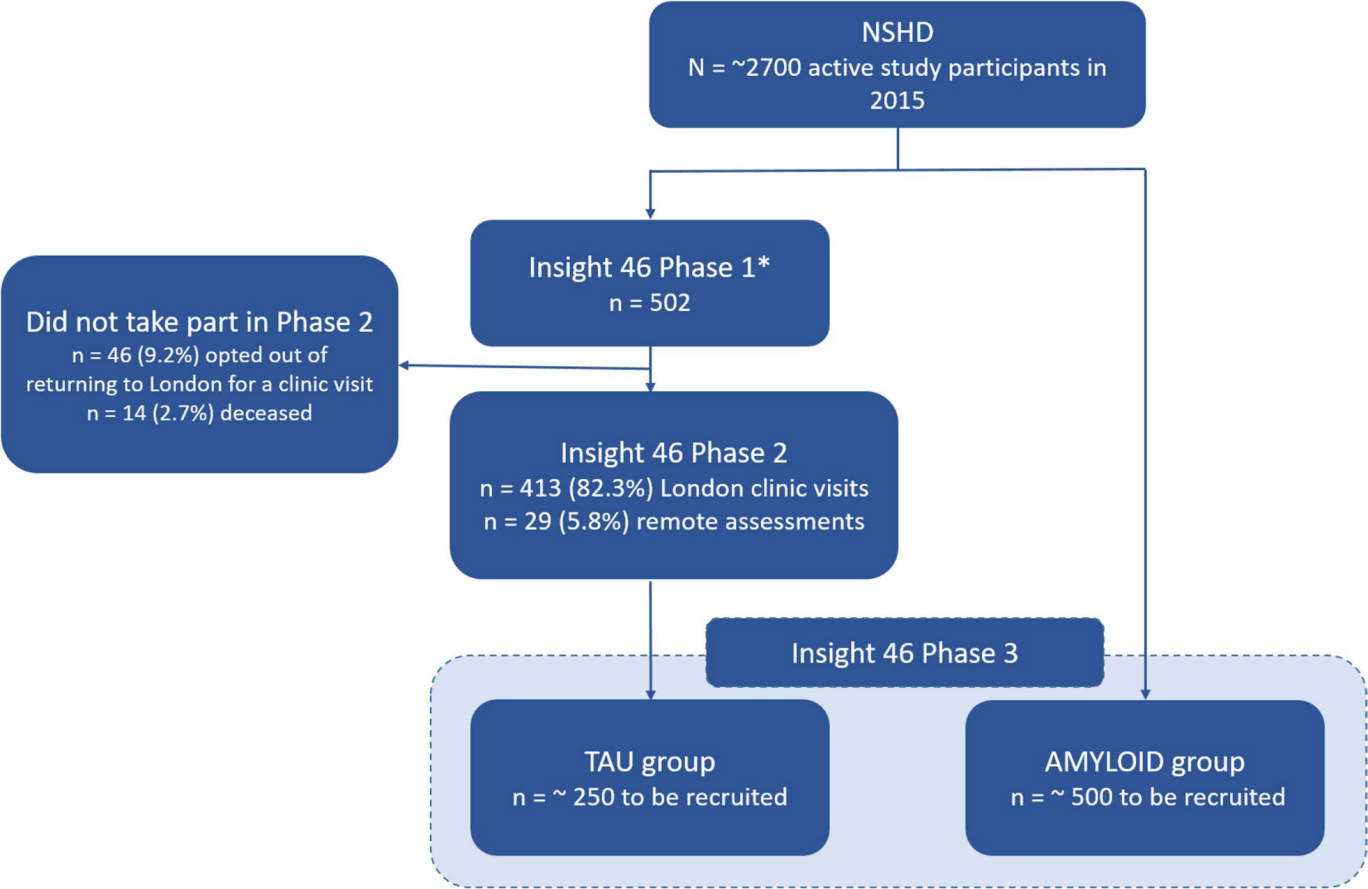
Overview of recruitment pathways for Insight 46. * Phase 1 recruitment rates have been previously described [14]

The two recruitment pathways for phase 3 of Insight 46 are shown in Fig. 1. The aim is to recruit 250 participants (the TAU group) for whom amyloid PET and MRI data are available from both phases 1 and 2 of Insight 46. Recruitment for this group will prioritise participants who had a lumbar puncture during phase 2 so that we can capitalise on their breadth of data. Furthermore, recruitment will prioritise individuals with evidence of amyloid pathology or accumulation based on phase 1 and 2 PET scans and who are therefore at increased risk of cognitive decline. We will also aim to recruit 500 participants (the AMYLOID group) who are members of the NSHD but who have not previously participated in Insight 46. Both groups will undergo the same clinical assessments in phase 3, with the exception that the TAU group will undergo PET scanning using the tau tracer [18F]MK-6240 (florquinitau) and the AMYLOID group with the amyloid tracer [18F]florbetaben (Neuraceq).

### Statistical considerations

The rates of ß-amyloid PET positivity found in phase 1 of Insight 46 (18.6%, using a standard uptake value ratio cut point for positivity of 0.61) are in line with predicted estimates in this population [15]. Our analyses to date [16–20] demonstrate that the Insight 46 sample size (n = 462 useable PET scans) provides more than sufficient power to investigate the relationship between ß-amyloid and a range of cognitive scores, cerebrovascular measurements, imaging outcomes, and blood- and CSF-based biomarkers. We expect the ß-amyloid positivity-rate in the phase 3 AMYLOID group to be at least as high as the TAU group, which will increase the power to explore more subtle relationships between life course outcomes and Insight 46 measurements.

## Clinical protocol description

### Overview of research visits

All non-imaging components of the Insight 46 clinic visits take place over a two-day period in assessment rooms at the Bloomsbury Centre for Clinical Phenotyping at University College London (UCL), London, UK. Multimodal neuroimaging data are acquired on a single 3T Siemens Biograph mMR combined PET/MRI scanner at UCLH Macmillan Cancer Centre. During phase 3 only, an additional MRI was acquired for each participant on a dedicated 3T Siemens MAGNETOM Prisma scanner at Chenies Mews Imaging Centre to enable more advanced acquisition techniques. A comparison of the data collected at each of the three phases of Insight 46 is provided in Table 2.

**Table 2:** Assessments administered during each phase of Insight 46

Assessment	References	Phase 1	Phase 2			Phase 3
			*Clinic*	*Remote – computer*	*Remote - telephone*	
**Neuropsychological Assessments**						
12-Item Face—Name Associative Memory Exam (FNAME)	[10, 18, 21]	✓ (version A)	✓ ^a^ (version A)	✓ ^a^ (version A)	–	✓ ^a^ (version B)
15-Item Word List Learning Task	[11, 22]	–	–	–	–	✓ ^a^
Adult Memory and Information Processing Battery (AMIPB) Complex Figure	[23]	–	✓ ^a^ (AMIPB figure version 1)	✓ ^a^ (AMIPB figure version 1)	–	✓ ^a^ (AMIPB figure version 2)
Choice Reaction Time/Response Inhibition	[10, 19]	✓	✓	‒	‒	✓ (inhibition condition removed, choice reaction time still administered)
Graded Naming Test (GNT)	[24]	–	✓	✓	–	✓
Instructionless Eye Tracking	[25]	✓ (first administered in Feb 2017)	✓	–	–	–
Irrelevant Distractor Task	[10]	✓	–	–	–	–
Visual Search Speed Task	[11, 22]	–	–	–	–	✓
Visual Short-term Memory Binding	[10, 26]	✓	✓	–	–	✓
Visuomotor Integration Circle-Tracing Task with Concurrent (Dual-Task) Serial Subtraction	[10, 27]	✓	✓	–	–	✓ (plus single-task circle tracing and single-task serial subtraction)
Wechsler Abbreviated Scale of Intelligence (WASI) – Matrix Reasoning	[10, 18]	✓	✓	✓	–	✓
Wechsler Adult Intelligence Scale-Revised (WAIS-R) – Digit-Symbol Substitution Test (DSST)	[10, 18]	✓	✓	✓	✓	✓
Wechsler Memory Scale – Revised (WMS-R) Logical Memory Recall IIa (Immediate and Delayed)	[10, 18]	✓	✓	✓	✓	✓
Wechsler Memory Scale (WMS) Digit Span Forward and Backward	[28]	–	–	–	–	✓
**Digital Technologies Used During Neuropsychological Assessments**						
Anoto AP-701 Digital Pen	[29]	–	–	–	–	✓ (DSST, AMIPB complex figure, MMSE)
Audio Recordings	[30]	–	✓ (GNT)	✓ (GNT)	–	✓ (GNT, WMS-R Logical Memory Recall IIa Immediate and Delayed, Serial Subtraction)
Empatica		–	✓	–	–	✓
**Memory and Clinical Interviews**						
AD8 Dementia Screening Interview	[10, 31–33]	✓	✓	✓	✓	✓
Clinical Dementia Rating (CDR) Scale	[34, 35]	–	✓	✓	✓	✓
Delirium Questionnaire		–	✓	✓	✓	✓
History of Neurological Diagnosis (Personal and Family)	[10, 33]	✓	✓	✓	✓	✓
Mini Mental State Examination (MMSE)	[10, 18, 36]	✓	✓	–	–	✓
MyCog	[10, 33]	✓	✓	✓	✓	✓
Subjective Cognitive Decline Questions	[10, 33]	✓	✓	✓	✓	✓
**Physical Assessments**						
Blood Pressure	[10, 37, 38]	✓ (lying, standing)	✓ (sitting, lying, standing)	–	–	✓ (sitting, lying, standing)
Bradykinesia Akinesia Incoordination (BRAIN) Tap Test	[10]	✓	✓	–	–	✓
Self-paced Gait in Isolation and with Cognitive Task	[10]	✓	✓	–	–	✓
Walking Speed	[39]	–	–	–	–	✓
Height	[10, 11]	✓	✓	–	–	✓
Weight	[10, 11]	✓	✓	✓ (estimated)	✓ (estimated)	✓
Hip and Waist Circumference	[11, 40]	–	–	–	–	✓
Bioimpedance		–	–	–	–	✓
Unified Parkinson’s Disease Rating Scale (UPDRS) – Part III: Motor Examination	[10]	✓	✓	✓ (partial)	–	✓
6-Minute Stepper Test	[13, 41]	–	✓	–	–	✓
Grip Strength	[11, 42]	–	–	–	–	✓
Chair Rises	[11, 43]	–	–	–	–	✓
Standing and Walking Balance	[11, 44]	–	–	–	–	✓
Spirometry	[45, 46]	–	–	–	–	✓
**Home Wearables**						
Actigraphy		–	✓	✓	✓	✓
Glucose Monitor	[47]	–	–	–	–	✓
**Sensory**						
Pure Tone Audiometry	[10, 48]	✓	–	–	–	–
Word in Noise	[10]	✓	–	–	–	–
University of Pennsylvania Smell Identification Test (UPSIT)	[10, 49]	✓	–	–	–	–
Portable Eye Examination Kit (PEEK)	[10]	✓	–	–	–	–
Spoons Taste Test	[50]	–	✓	–	–	–
**Self-Complete Questionnaires**						
Dental Hygiene	[10]	✓	–	–	–	–
Handedness	[10]	✓	–	–	–	–
State-Trait Anxiety Inventory (STAI)	[10, 33]	✓	✓	✓	✓	✓
Question About Movement in Sleep	[10]	✓	–	–	–	–
Epworth Sleepiness Scale (ESS)	[51, 52]	–	✓	✓	✓	✓
Pittsburgh Sleep Quality Index (PSQI)	[53]	–	✓	✓	✓	✓
REM Sleep Behaviour Disorder Questionnaire (RSBDQ)	[54]	–	✓	✓	✓	✓
Perseverative Thinking Questionnaire (PTQ)	[55]	–	✓	✓	✓	✓
Head Injury Questionnaire	[56]	–	✓	✓	✓	✓
General Health Questionnaire (GHQ)-28	[11, 57, 58]	–	–	–	–	✓
**Biofluid Sampling**						
Blood – Haematology and Biochemistry	[59]	✓	–	–	–	✓
Blood – DNA		✓	–	–	–	–
Blood – RNA		–	✓	–	–	✓
Blood – Cell Lines	[60, 61]	–	✓	–	–	✓
Blood – Biomarkers	[16, 62]	✓	✓	–	–	✓
Urine – Biomarkers		✓	✓	–	–	✓
Urine – Spot Urine Sample	[11]	–	✓	–	–	–
Cerebrospinal Fluid	[62]	–	✓	–	–	✓
**Cardiovascular Measurements**						
2D and 3D Echocardiography	[11, 13]	–	✓	–	–	✓
Carotid Intima-Media Thickness	[11, 13]	–	✓	–	–	✓
Carotid Femoral Pulse Wave Velocity (PWV)	[11, 13]	–	✓	–	–	✓
Pulse Wave Analysis (PWA) – Central Blood Pressure	[11, 13]	–	✓	–	–	✓
Electrocardiogram (ECG)	[11, 13]	–	✓	–	–	✓
Functional Near-Infrared Spectroscopy (fNIRS)	[13]	–	✓	–	–	✓
**Imaging**						
Positron Emission Tomography (PET)	[63]	[^18^F]florbetapir (Aβ)	[^18^F]florbetapir (Aβ)	–	–	[^18^F]florbetaben (Aβ) or [^18^F]MK6240 (tau)
Brain Magnetic Resonance Imaging (MRI)	[63, 64]	✓	✓	–	–	✓
Dual-energy X-ray Absorptiometry (DXA)	[11, 40]	–	–	–	–	✓
Optical Coherence Tomography (OCT)		–	–	–	–	✓

^a^These assessments were part of an accelerated-forgetting paradigm that involved calling participants seven days after their research visit to ask follow-up questions

**Fig. 2:**
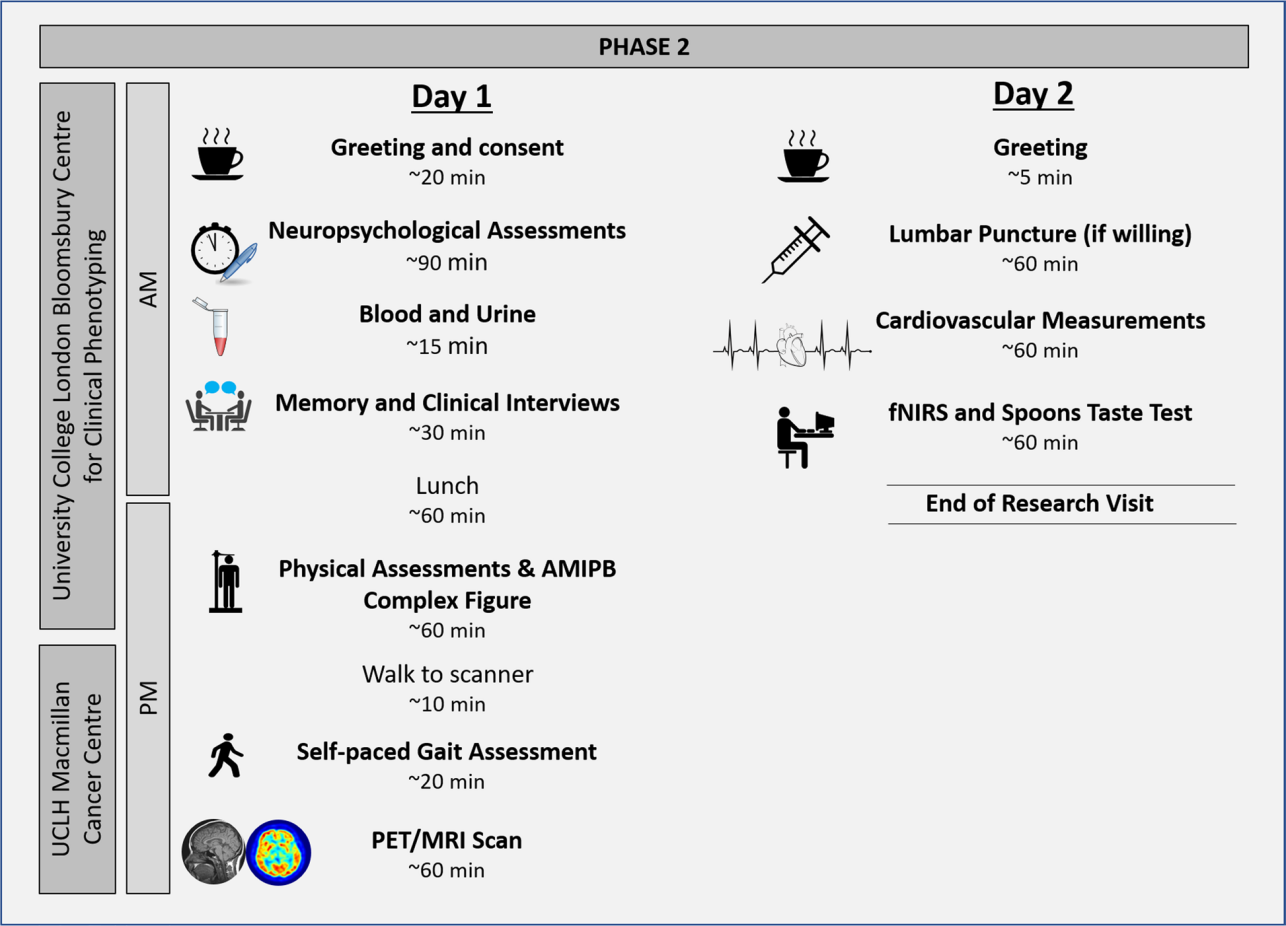
Overview of days 1 and 2 of a typical phase 2 research visit. Breaks were provided to the participants at multiple points throughout the day. If the participant consented to a lumbar puncture, the blood and urine were collected on day 2 at the same time as the cerebrospinal fluid. The self-complete questionnaires and the discussion about the actigraphy device were incorporated at different times during the research visit depending on how slowly or quickly the day progressed

**Fig. 3:**
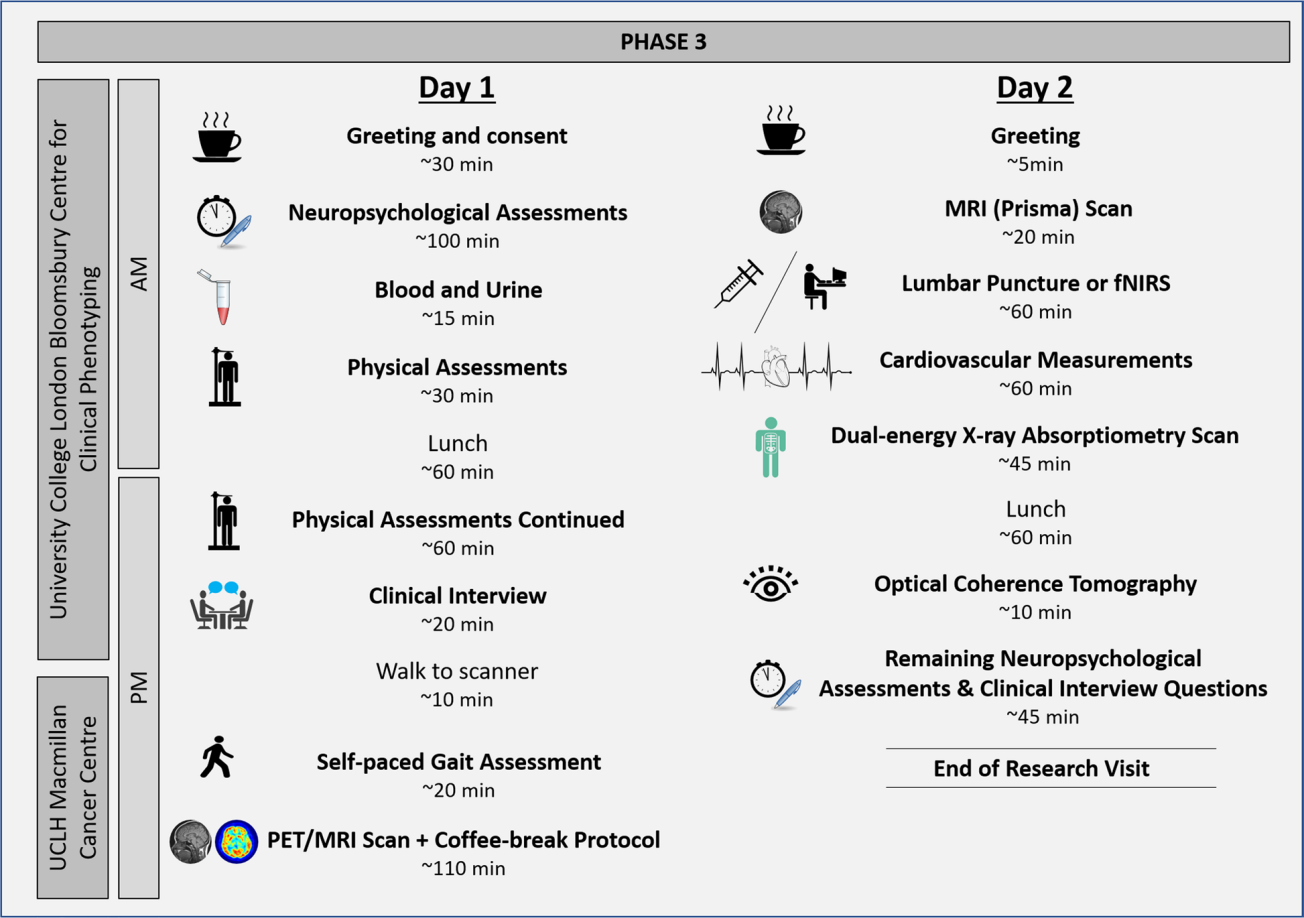
Overview of days 1 and 2 of a typical phase 3 research visit. Breaks are provided to the participants at multiple points throughout the day. If the participant consents to a lumbar puncture, the blood and urine are collected on day 2 at the same time as the cerebrospinal fluid. The self-complete questionnaires and the discussion about the wearable devices are incorporated at different times during the research visit depending on how slowly or quickly the day progresses

An outline of a typical research visit for phases 2 and 3 are depicted in Figs. 2 and 3, respectively. During phase 2, 413 participants completed the day 1 assessments for which they were eligible, and 401 participants (97%) also took part in the day 2 assessments. Although the timings and order of the assessments occasionally change due to scheduling conflicts, staffing shortages, and other logistical issues, there are some parts of the visit that rarely or never change. For example, all efforts are made to ensure the neuropsychological assessment block is completed during the morning of day 1; lumbar punctures are always completed during the morning of day 2 followed by the cardiovascular assessments so that the participants are able to rest for one hour; blood and urine are almost always collected during the mornings unless an issue with collection arises; and the PET/MRI is always completed in the late afternoon due to the timing of the PET tracer production.

Due to the COVID-19 lockdown measures across the UK, the phase 2 study protocol transitioned from face-to-face data collection to remote testing methods to allow the ongoing collection of longitudinal data. Of the 48 participants due to be invited to a phase 2 research visit, 29 consented to complete a remote research assessment, as shown in Table 1. Participants with a computer or tablet with a webcam, microphone, and screen dimensions greater than nine inches were offered a Microsoft Teams videoconference assessment; those without were offered assessments via telephone to ensure maximum data collection.

### Description of assessments added to the study protocol

The sections below provide information about assessments that make up the Insight 46 study protocol that have not been described elsewhere (i.e., a detailed description of the phase 1 protocol [10] and details about the cardiac assessments and fNIRS [13]) or assessments that have been substantially altered since described in those publications.

#### Neuropsychological assessments

**Fig. 4:**
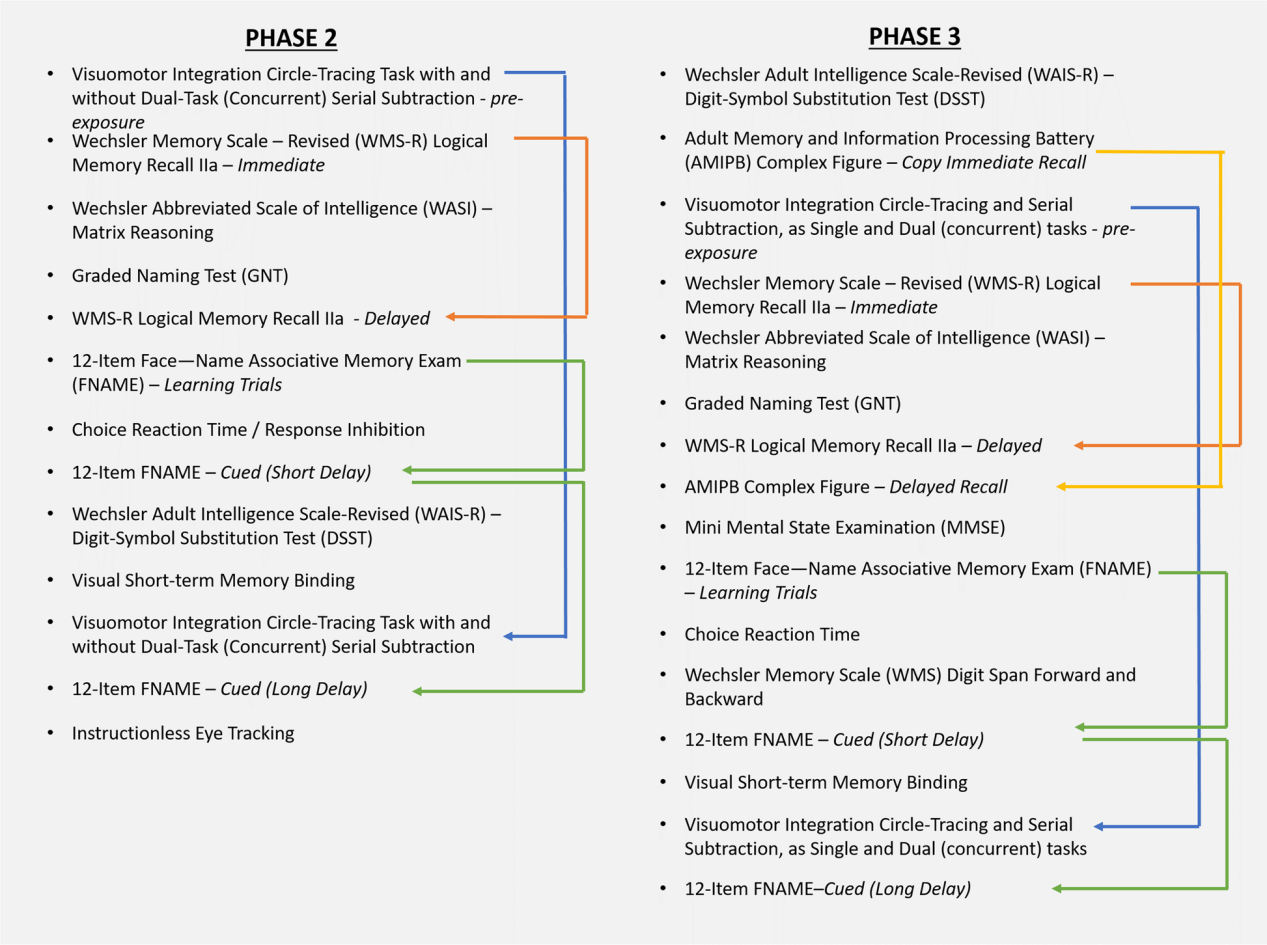
Tests administered during the neuropsychological assessment block of Insight46 phases 2 and 3. Tests with multiple sections (e.g., immediate and delayed recall) are linked by a coloured line

Figure 4 provides a list of the tests that are administered as part of the main neuropsychological assessment block during phases 2 and 3. With few exceptions, this assessment block is administered during the morning of day 1 to reduce participant fatigue. All efforts have been made by the research team to ensure test administration continuity between the phases of data collection. The order of the test administration presented in Fig. 4 was only altered if an issue arose with the equipment or the participant requested a break.

The 15-item word list learning task and the visual search speed task (letter cancellation) were added to phase 3 to measure verbal memory and processing speed [65]. These assessments were performed as part of NSHD data collections at ages 43, 53, 60–64, and 68–69 [66, 67]. The word list task consists of 15 words that are presented serially to the participant for approximately two seconds each. Participants are given one minute to write down as many words as they can recall. This procedure is repeated for a total of three trials. After a delay of approximately 90 s (during which the visual search task is completed), the participants are given another minute to write down as many words as they can recall. The visual search task requires participants to cross out the letters P and W, which are randomly embedded within a page of other letters, as quickly and accurately as they can within one minute.

The Adult Memory and Information Processing Battery (AMIPB) complex figure drawing is a test of visual memory recall [68]. This was administered for the first time in phase 2. In phase 3, a different version of the complex figure is administered to mitigate practice effects for the participants in the TAU group who had performed this task previously. Participants are asked to copy a complex figure as accurately as possible. The figure and copy are then removed and participants are immediately asked to draw the figure again from memory. After a 30-min delay in which participants complete other cognitive assessments, participants are asked to draw the figure again from memory.

The choice reaction time test has been described previously [10, 19]. In phase 3, the response inhibition condition was removed.

The Graded Naming Test (GNT) is administered in phases 2 and 3, and it measures naming ability for objects graded for their difficulty [24]. Participants are shown 30 black and white line images, starting with common objects, before moving to less common objects. Participants are asked to name each item aloud. The task is audio recorded to measure response latency. An automated software-based solution was developed using the Speech Filing System (SFS) [69] to extract temporal information about the onset of voicing.

The instructionless eye tracking test was introduced during phase 1 (*n* = 179) and continued to be administered in phase 2. This is a free-viewing test designed to assess multiple cognitive domains, including recognition memory, semantic processing, and social interaction. Participants are shown 48 images for three seconds each, and their eye movements are recorded using a desk-mounted video-based eye tracker (Eyelink 1000 plus) at 100 Hz [25]. Participants are not given any explicit instructions other than to look at the screen. The stimuli are divided into four groups: i) scene exploration, which contains 28 images of social scenes, social interactions, and missing items; ii) a spatial anticipation task (based on the Brixton test), which assesses participants’ ability to detect and follow rules in sequences of stimuli [70]; iii) ten sentences, where half the sentences are semantically congruent and half semantically incongruent; and iv) a recognition memory test of ten pairs of images, one of which is a new image and the other an image shown previously in the social interaction task.

The visuomotor integration circle-tracing task, with concurrent (dual-task) serial subtraction, has been described in detail [10, 27]. The protocol remained unchanged from phase 1 to phase 2; however, in phase 3, additional trials were added to measure individual aspects of the tasks. Participants are asked to perform the circle-tracing and serial subtraction tasks independently (as single tasks). This will allow performance to be compared between single- and dual-task conditions and to investigate dual-task cost. All subtraction trials are audio-recorded.

The digit span forward and backward task from the Wechsler Memory Scale–Revised (WMS-R) was added to phase 3 to capture verbal working memory. Participants are asked to repeat increasingly longer strings of numbers exactly as presented (forward condition) and in reverse order (backward condition) [28].

Accelerated long-term forgetting [71] is assessed during phases 2 and 3. Participants are given a sealed envelope at the end of the research visit. They are asked to have this envelope to hand and to open as instructed during a telephone call that is scheduled seven days later. During the call, participants are:

- asked to draw the AMIPB complex figure from memory.
- given a complex figure recognition memory test with four trials and asked to identify the correct part of the original figure from three similar options.
- asked questions about names, faces, and occupations from the 12-Item Face-Name Associative Memory Exam (FNAME).

For phase 3 only, participants are also asked about the 15-item word list described above. During the call, participants are given one minute to write down as many words as they can remember and immediately seal the list in an envelope to be returned to the study team.

#### Digital technologies used during neuropsychological assessments

In phase 3, the Anoto AP-701 digital pen is incorporated for use in all pen and paper tasks: the Digital Symbol Substitution task (DSST), AMIPB complex figure, and the sentence and pentagon sections of the Mini Mental State Examination (MMSE). The Anoto AP-701 is commercially available and combines an ordinary ballpoint pen with a pressure sensor and a specialised camera that captures its coordinates on a page approximately 75 times per second, with a spatial resolution of ± 0.002 inches. These timestamped coordinates constitute a digital record of the drawing from which various metrics can be derived (e.g., number and order of strokes, “think time” vs. “ink time”), providing insights into how a task is performed [29].

Audio recordings are used in phases 2 (during the GNT) and 3 (during the GNT, Wechsler Memory Scale–Revised (WMS-R) Logical Memory Recall IIa immediate and delayed conditions, and serial subtraction). The main goals of the audio recordings are to maximise scoring accuracy (by allowing raters to listen back to responses) and to analyse other aspects of responses from which potentially sensitive cognitive measures can be derived, such as word choice (allowing classification of errors), response latencies, and speech-based metrics (e.g., fluency, tonal quality, and pitch) [30].

To measure physiological arousal during the cognitive assessments in phases 2 and 3, participants are asked to wear an Empatica E4 wristband on both wrists throughout the cognitive testing session (https://www.empatica.com/e4-wristband). This is a medical grade (CE-certified) wearable device that measures electrodermal activity (EDA), temperature, acceleration, and photoplethysmography (PPG), which derives heart rate and inter-beat interval (IBI) from the blood volume pulse (BVP). These real-time data may provide insights into how factors such as anxiety or stress can affect performance on cognitive testing. The wristband is the size of a small watch and does not impede movement of the hands.

#### Clinical interviews

The Clinical Dementia Rating (CDR) is a global scale designed to assess the severity of dementia [35] using a semi-structured interview with the participant and an informant who knows the participant well. The CDR is administered during phases 2 and 3 to rate the participant’s cognitive performance in six domains: memory, orientation, judgement and problem solving, community activities, home and hobbies, and personal care. A global CDR is determined by combining the scores from each domain in accordance with an established scoring algorithm. All assessors undertake a standardised CDR training program online.

A delirium questionnaire is administered during phases 2 and 3 and consists of a participant and an informant interview. The participant is asked to think about a time that they have been unwell, particularly if admitted to the hospital, and to state whether they have experienced any of the following symptoms over the course of a few hours or days: new or worsening confusion or disorientation, uncharacteristic drowsiness, agitation, aggression or violence, hallucinations, or thinking clearly but then more muddled. If the participant answers yes to any of the above, further questions probe which illness led to this, what was the approximate duration, and the year the symptoms occurred. A similar set of questions is used to obtain a history of delirium affecting the participant from the informant.

#### Physical assessments

Lying and standing blood pressure are measured during each phase of Insight 46 using an Omron HEM-907 automated digital oscillometric sphygmomanometer and an appropriately sized cuff. Seated blood pressure, which was added to phase 2 and continued during phase 3, is assessed prior to lying and standing blood pressure.

The six-minute self-paced stepper test [41] is administered during phases 2 and 3 to assess the participant’s exercise capacity and the effects of exercise on physiological measures, including blood pressure (both phases 2 and 3) and heart rate (phase 3 only). The test is performed while the participant is wearing an accelerometer (LPMS-B Intertial Measurement Unit; Life Performance Research Inc.) on the lower back to assess movement. Participants are instructed to perform as many steps as they can at a pace that they can maintain for six minutes. Blood pressure is measured with a Tango M2 SunTech device at rest; at two, four, and six minutes during exercise; immediately post-exercise; and at a three-minute recovery. The number of steps completed by the participant, their duration of exercise, and their perceived level of exertion on a modified (0–10) Borg scale are recorded [72].

Four measures of physical capability (chair rising, standing balance, grip strength, and walking speed) are obtained in phase 3 using standardised protocols as described elsewhere [39]. In brief, chair rise time is measured as the time taken for the participant to rise from a chair and sit back down again ten times as quickly as possible. Standing balance is measured as the duration that the participant can stand on one leg, first with their eyes open and then with their eyes closed, up to a maximum of 30 s. Grip strength is determined using an electronic handgrip dynamometer (Jamar Plus), twice in each hand. Walking speed in metres per second is derived from the time taken for the participant to walk 2.44 m (8 feet) at their usual pace.

Lung function—measured as forced expiratory volume in one second (FEV1) and forced vital capacity (FVC)—is obtained in phase 3 using a spirometer (Easy on-PC sensor) [45, 46]. Participants are asked to stand and to take as deep a breath as possible to fill their lungs to capacity. They are then asked to seal their lips around the spirometer tube, place their tongue under the mouthpiece, and blow out as hard and fast as possible until no more air can come out of their lungs. Measurements are repeated until three similar readings are obtained, up to a maximum of five times.

Bioimpedance measurements are collected in phase 3 using a Tanita BC 418 body composition analyser (TANITA Cooperation, Japan). This provides measurements of body fat ratio, body fat mass, fat-free mass, estimated muscle mass, and base metabolic rate. Waist and hip circumference are also measured in phase 3 [11, 40].

#### Home wearables

To quantify sleep and circadian measures during phases 2 and 3, participants are asked to wear a wrist actigraphy device for seven days following their clinic visit (Philips Actiwatch Spectrum Pro). The device detects light and motion, allowing periods of rest and activity to be monitored, and it can be used to make inferences about sleep and wakefulness. To complement actigraphy data, participants are also asked to complete a sleep diary, documenting their self-reported sleep and waking times.

During phase 3, participants are asked to wear a FreeStyle Libre Pro IQ Sensor glucose monitor for seven days following their clinic visit [47]. The monitor provides a continuous measure of blood glucose levels recorded every 15 min.

#### Sensory

As previously described, hearing and smell tests were administered during phase 1 [10, 49, 73]. During phase 2, a novel sensory test—a spoons taste test—was administered. Participants are asked to taste spoons that are made from different materials (stainless steel, silver, gold, copper, tin, and zinc), which have different metallic and bitter tastes [50]. The order in which the spoons are presented to each participant is randomized. The participants are then asked to place the spoons in order according to how metallic, bitter, salty, sweet, and unpleasant they found the tastes of each spoon.

#### Self-complete questionnaires

The study participants are asked to complete a series of questionnaires in phases 2 and 3 that relate to the topics of sleep, head injuries, repetitive negative thinking, and general health. All questionnaires are completed during the research visit.

Three sleep questionnaires are administered: Epworth Sleepiness Scale (ESS) [51, 52], Pittsburgh Sleep Quality Index (PSQI) [53], and REM Sleep Behaviour Disorder Questionnaire (RSBDQ) [54].

The ESS provides a measure of the participant’s general level of daytime sleepiness [51]. The questionnaire lists eight situations, and the respondent rates how likely they are to doze off or fall asleep in each of those situations. The scores for each situation are summed, giving a total score between zero and 24. A total score greater than 16 indicates a high level of daytime sleepiness.

The PSQI is designed to measure sleep quality and assess sleep disturbances during the past month [53]. With permission from the author, the term “room mate” within the questionnaire was changed to “housemate” in keeping with UK norms. Participants are asked to provide responses based on experiences over the majority of days and nights during that one-month period. The questionnaire is divided into eight domains: sleep quality, sleep latency, sleep duration, habitual sleep efficiency, sleep disturbance, use of sleep medication, and daytime dysfunction. The scores from each domain are summed to create a global PSQI score. A global score less than or equal to five is associated with good sleep quality, while a score greater than five is associated with poor sleep quality.

The RSBDQ is a ten-item questionnaire designed to detect clinical features of REM sleep behaviour disorder, with one of the items having four parts [54]. Participants are asked to tick either “yes” or “no” in response to each statement in relation to their current sleep behaviours. Each “yes” answer is worth one point, resulting in a total score between zero and 13. A score of five or more is indicative of REM sleep behaviour disorder.

The study team created a questionnaire that is administered during phases 2 and 3 to gather information about head injury. The participants are asked:

- whether they had been admitted to the hospital following a head injury (including frequency and age);
- whether any head injuries involved heavy bleeding into or around the brain, bruising of brain tissue, a penetrating head injury, or a brain stem injury;
- whether the head injury involved a loss of consciousness and, if so, the frequency, age, and whether the loss of consciousness was more or less than 30 min;
- whether they experienced blurred vision, confusion, headache, nausea, or a feeling of daze or dizziness (including frequency and age); and
- whether they played rugby, football or boxed consistently for at least a year (including frequency at peak performance and ages).

One year into phase 2, the Perseverative Thinking Questionnaire (PTQ) [55] was introduced. The PTQ consists of 15 statements that aim to assess the core characteristics of repetitive negative thinking: repetitiveness, intrusiveness, and difficulties with disengagement. Participants are asked to use a scale from zero (never) to four (almost always) to rate the extent to which each statement applies when thinking about negative experiences or problems. The scores are summed to create a total score between zero and 60. Three subscores are also generated, representing core characteristics of repetitive negative thinking, unproductiveness of repetitive negative thinking, and repetitive negative thinking capturing mental capacity.

The final self-complete questionnaire is the General Health Questionnaire (GHQ)-28 [57, 58], which is used to gain a better understanding of the participant’s emotional health over recent weeks. The GHQ-28 was introduced in phase 3 and consists of 28 questions divided equally into four categories: measurement of somatic symptoms, anxiety and insomnia, social dysfunction, and depression. Participants are asked to respond to each question on a Likert scale of 0, 1, 2, or 3 [58]. The individual scores are then summed to create a total score between zero and 84. The GHQ-28 was administered during previous waves of data collection for the NSHD [74, 75], allowing longitudinal analyses.

#### Biofluid sampling

In addition to continuing to collect blood (plasma, serum, and Guthrie cards) and urine samples for biomarker exploration as in phase 1 [10], cerebrospinal fluid (CSF) samples are also being obtained for this purpose from phase 2 onwards if the study participant is willing and eligible to undergo a lumbar puncture. Contraindications to lumbar puncture include neuroimaging evidence of a space-occupying lesion with mass effect, tonsillar herniation due to Chiari malformation, or increased intracranial pressure; known bleeding disorder or use of anticoagulation/antiplatelet medication (except aspirin 75 mg once daily); congenital spine abnormality; lignocaine allergy; or active rash over the proposed puncture site. During consent, participants are informed of possible side effects, including headache (1 in 10), backache, infection or bleeding, and nerve damage (very rare). With the participant in the lateral decubitus position or seated with lumbar forward flexion, an intervertebral space between L2/L3 and L5/S1 is first located by palpation. The skin is then prepared with chlorhexidine 2%/ethanol 70% solution, before local anaesthesia (lignocaine 2%, maximum 3 mg/kg) is administered. An atraumatic 22G spinal needle is then inserted into the anaesthetised space and CSF is collected by passive drip into two polypropylene tubes, aiming for a total of 20 ml. An aseptic technique (sterile gloves and field) is maintained throughout. The CSF samples are then transported on ice to the laboratory where they are centrifuged (1750 g/ 3000 rpm for 10 min at 4 °C) and aliquoted into up to 40 polypropylene cryovials, before being stored in a -80 °C freezer within 60 min of arrival. Planned biomarker analyses in plasma and CSF include neurofilament light chain, Aβ40 and Aβ42, glial fibrillary acidic protein (GFAP), and several phosphorylated tau moieties, as well as novel biomarkers for synaptic function (including SNAP-25 and β-synuclein).

During phases 2 and 3, blood for RNA analysis is collected into a 2.5 ml PAXgene tube and stored at -80 °C for later processing and RNA extraction. Blood samples are also being collected during phases 2 and 3 for the generation of cell lines, with the goal of generating induced pluripotent stem cells from individuals with low/high risk of disease. The blood is collected into an 8 ml cell preparation tube (CPT) and kept at room temperature, before being transported to the laboratory within 24 h of collection. Peripheral blood mononuclear cells (PBMCs) are extracted using the Ficoll-Paque protocol and cryopreserved for future reprogramming to induced pluripotent stem cells [60, 61].

Similar to phase 1, blood samples are being collected for clinical measures in phase 3. One 4 ml EDTA sample is collected to measure haemoglobin, platelet count and haemoglobin A1c (HbA1c). One 5 ml SST sample is collected to measure urea, creatinine, vitamin B12, folate, vitamin D (25-OH), random glucose, and thyroid-stimulating hormone (TSH).

In phase 2 only, participants are asked to provide a mid-stream early morning urine sample in a 25 ml urine collection pot for dried urine spot processing. The urine is pipetted onto four circles (each holding 25–80 µL) on Whatman 903 protein saver cards and allowed to dry at room temperature for 3–4 h before storage in a zip-lock plastic bag with desiccant at -80 °C. The remaining urine is aliquoted into three × 2 ml samples and stored at -80 °C. Metabolomic analyses are planned, with a view to validating biomarkers and studying the metabolism of drugs.

#### Brain imaging

Phases 1 and 2 of the study followed the same brain imaging protocol design, as previously described [10], with the acquisition of imaging data on the Siemens Biograph mMR 3T PET/MRI scanner at the UCLH Macmillan Cancer Centre. For phase 3, the brain scanning takes place on both the PET/MRI at UCLH and the Siemens Prisma 3T MRI at Chenies Mews Imaging Centre, which enables the use of more advanced acquisition techniques due to better sensitivity and hardware performance. The advanced MR sequences acquired on the Prisma are as follows:

• magnetization-prepared 2 rapid acquisition gradient echo (MP2RAGE) [76], which provides quantitative T1 maps in addition to high-contrast T1-weighted morphological images.
• multi-delay pseudo-continuous arterial spin labelling (PCASL) [77], which enables quantitative estimation of cerebral blood flow (CBF) and arterial transit time (ATT) (two indicators of cerebrovascular health). Angiographic scout images are used to ensure appropriate and accurate placement of the labelling plane for each participant.
• inversion recovery intravoxel incoherent motion (IR-IVIM) [78], to estimate perfusion volume fraction and parenchymal diffusivity without contamination from cerebrospinal fluid (CSF).

Table 3 provides the basic parameters for the MP2RAGE, PCASL, and IR-IVIM sequences acquired on the Siemens Prisma 3T MRI.

**Table 3:** Details of sequences acquired on the Siemens Prisma 3T MRI

	**MP2RAGE**	**Multi-delay 3D GRASE PCASL**	**IR-IVIM**
**Voxel resolution (mm**^**3**^**)**	1 × 1 × 1	3.5 × 3.5 × 4.5	2.4 × 2.4 × 2.4
**Matrix size**	224 × 216 × 176	64 × 56 × 32	88 × 88 × 45
**FoV (read x PE) (mm)**	224 × 216	224 × 196	210 × 210
**Slice coverage (mm)**	176	144	130 (20% slice gap)
**Orientation**	Sagittal	Transverse	Transverse
**PE direction**	A ≫ P	R ≫ L	P ≫ A
**TE (ms)**	2.59	13.44	72
**TR (ms)**	4000	PLD + 2200	10,700
**Flip angle (°)**	4, 5	90 (excitation), 160 (refocusing)	90 (excitation), 180 (refocusing)
**Acquisition bandwidth (Hz/pix)**	220	2298	2030
**Parallel imaging**	× 3	None	× 2
**Total scan time**	5 min 38 s	6 min 16 s	4 min 09 s
**Other sequence-specific parameters**	TI1 = 600 ms	LD = 1800 ms	Slice-sel. IR EPI with TI = 1800 ms
	TI2 = 1800 ms	PLD = 400, 800, 1200, 1600, 1800, 2000, 2200, 2400, 2800	Single direction DWI (DW along PE) with b values: 0, 10, 20, 30, 40, 50, 60, 100, 200, 300, 400, 500, 600, 800, 1000 s/mm^2^
	PE partial Fourier 7/8	Additional M0 scan, with blip-reversed PE for distortion correction	b = 0 volume acquired with blip-reversed PE for distortion correction
		Background suppression	
		4 segments (in PE direction)	
		Slice partial Fourier 6/8	
		PCASL labelling plane positioned using sagittal and coronal neck ToF MRA	

*Abbreviations*: *DWI* Diffusion-weighted imaging, *EPI* Echo-planar imaging, *FoV* Field of view, *IR-IVIM* inversion recovery intravoxel incoherent motion, *LD* Labelling duration, *MP2RAGE* Magnetization-prepared 2 rapid acquisition gradient echo, *PCASL* Pseudo-continuous arterial spin labelling, *PE* Phase encoding, *PLD* Post-labelling delays, *TE* Echo time, *TI* Inversion time, *ToF* Time of flight, *TR* Repetition time

**Fig. 5:**
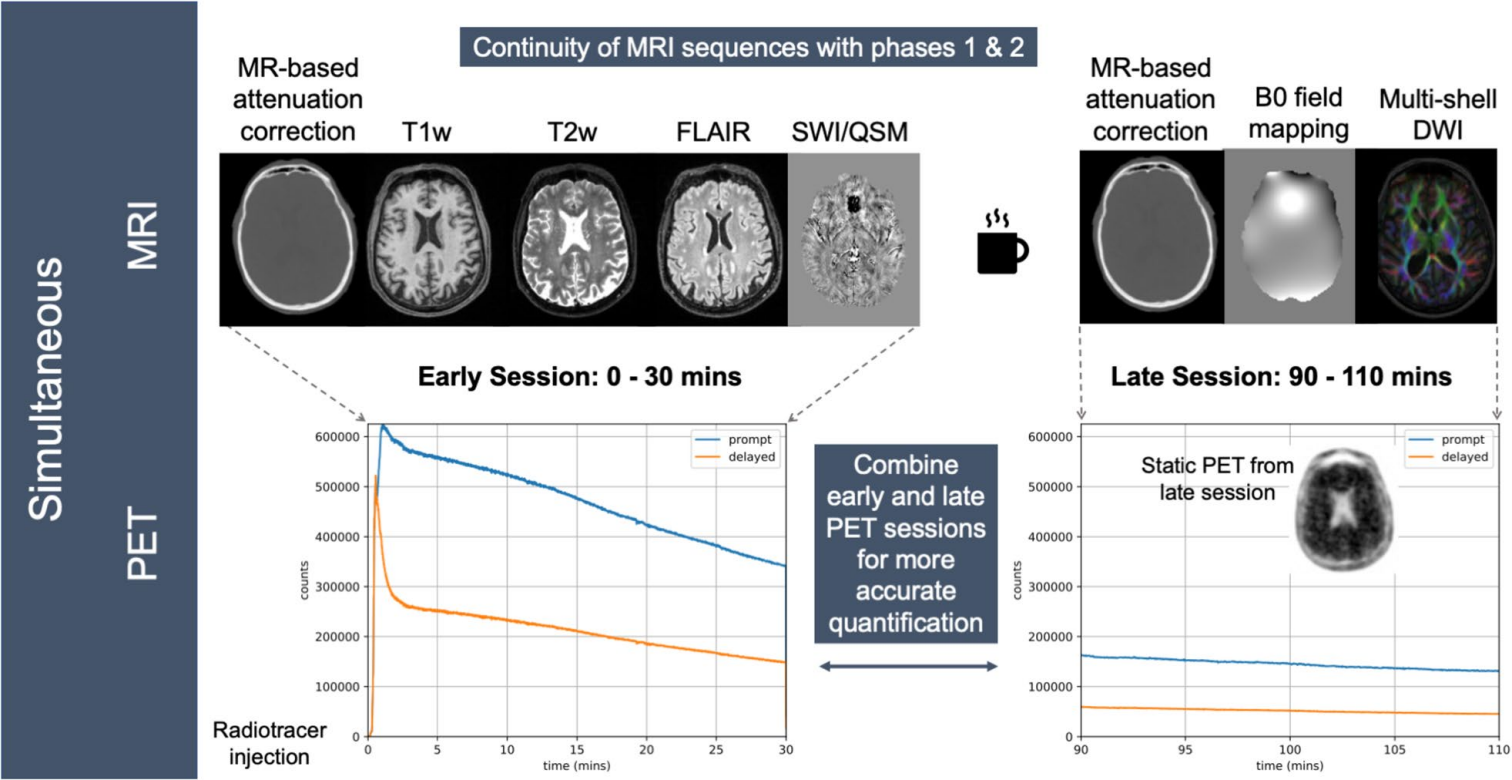
Diagram outlining the dual-time window (or coffee-break) PET-MR protocol used in Insight 46 phase 3. PET and MRI data are acquired simultaneously in early (0–30 min) and late (90–110 min post-injection) sessions. PET data are acquired in list-mode format. Example 'head-curve' plots generated with NiftyPET software are displayed, showing the total number of prompt and delayed counts detected per second across the early and late sessions. Above the plots are example images for MRI sequences acquired in each session, as well as the late session static PET image. Abbreviations: DWI, diffusion weighted imaging; FLAIR, fluid attenuated inversion recovery; MRI, magnetic resonance imaging; PET, positron emission tomography; SWI, susceptibility weighted imaging; QSM, quantitative susceptibility mapping

The PET/MRI imaging protocol has been revised for phase 3 to reflect the use of a different Aβ PET ligand—[^18^F]florbetaben (300 MBq) —compared to the one used in phases 1 and 2, as well as the use of the tau PET ligand [^18^F]MK-6240 (185 MBq). These two tracers require longer post-injection times for accurate quantification compared to [^18^F]florbetapir. A coffee-break protocol [79], see Fig. 5, is used to acquire PET data from 0–30 min and between 90–110 min post PET ligand injection. During the 0–30 min period, the following MRI sequences are acquired: volumetric T1, volumetric T2, volumetric FLAIR, and susceptibility-weighted imaging and quantitative susceptibility mapping. Participants are removed from the scanner during the 30–90 min period. During the 90–110 min period, multi-shell diffusion-weighted imaging and B0 field mapping are acquired. For phase 3, resting state fMRI data are not acquired, and the phase 1 and 2 arterial spin labelling (ASL) protocol is not run on the PET/MRI scanner because a more advanced multi-PLD ASL protocol has been implemented on the 3 T Prisma (see above). For all phases of Insight 46, PET data are acquired continuously during and following injection, apart from during the coffee-break, allowing the PET ligand dynamics to be assessed.

#### Other imaging

Dual-energy X-ray absorptiometry (DXA) scans were undertaken during the NSHD clinic visit at ages 60–64 [80], and DXA scans using a Hologic Bone Densitometer QDR Horizon system were added to phase 3 of Insight 46. The DXA scan assesses bone density, body composition (lean and fat mass), and aortic calcification, and it complements the other anthropometric and body composition measurements described above. The protocol includes a scan of the left hip (or right hip if the left is not possible), a scan of the lumbar spine, instant vertebral assessment (IVA), and a whole-body examination.

Optical coherence tomography (OCT)—a non-invasive eye scan that provides detailed retinal images, including images of small blood vessels—has also been added to phase 3. During the procedure, a trained technician first carries out an assessment of visual acuity in each eye, with appropriate spectacle and pinhole correction. The technician then completes the retinal imaging and OCT measurements for each eye using a Topcon DRI Triton device. The protocol includes fundus photography with fluorescence turned off, 3D OCT of the macula, 3D OCT of the optic nerve and disc, and angiography of the macula (4.5 × 4.5 mm).

### Duty of care

The duty-of-care protocol for phase 1 of Insight 46 has been described previously [10, 59]. That protocol included a description of information that was fed back to the participant and their general practitioner (GP) for anthropomorphic measures (height and weight); recumbent blood pressure; audiometry; and the results of a range of standard clinical blood tests (haemoglobin, platelet count, vitamin B12, urea, creatinine, random glucose, and TSH). That protocol also described the procedure for reporting any clear evidence of significant cognitive impairment or clinically detectable parkinsonism in previously undiagnosed individuals and the procedure for review of T1, T2, and FLAIR volumetric MRI sequences by consultant neuroradiologists. A summary of incidental findings detected on brain imaging and blood tests during phase 1 has been published [59].

As additional assessments have been incorporated into phases 2 and 3 of Insight 46, the duty-of-care protocol has been updated accordingly. For the newly added assessments described below, the participant’s GP is informed whether the participant consented to participate in the procedure, and feedback is given to the participant and their GP in accordance with the MRC/Wellcome Trust guidelines for feeding back health-related findings in research [81].

For the lung function assessment, the highest FEV1 and FVC readings taken with a spirometer are reported to the GP and participant. If the FEV1 divided by the FVC is less than 0.7, the participant is advised to visit their GP within one month.

GPs are informed if a participant consents to a lumbar puncture (LP), but no results are sent to the study participant or their GP as no routine testing of samples is performed. One day after the procedure, the participant is contacted to ascertain whether they have developed a post-LP headache or any other adverse event. For a typical postural post-LP headache, bed rest is advised until the pain subsides. Participants are contacted daily until the headache abates; if it persists by day seven, arrangements are made for a blood patch, although to date this has been required in only one case in phase 2. For back pain, the participant is advised to take over-the-counter analgesia if needed.

Duty-of-care guidelines for the 12-lead resting electrocardiogram (ECG), five-minute heart rate variability (HRV) recording, resting transthoracic echocardiogram, and common carotid artery intima-media thickness (cIMT) assessments have been modified from the previous 2006–2010 reporting used by the NSHD [13]. All these cardiac assessments are reviewed in real-time by a trained technician. In the event of an urgent and actionable finding (see Table 4 for examples), the technician immediately contacts a consultant cardiologist to discuss the action to be taken. Non-urgent findings are reviewed by a consultant cardiologist and, if appropriate, reported to the participant’s GP and to the participant telephonically within six weeks of the research visit. Table 4 lists examples of non-urgent incidental findings. Of note, the carotid ultrasound is performed for analysis of the common carotid intima media thickness, and it is beyond the scope of the study to determine the degree of carotid bifurcation stenosis.

**Table 4:** Incidental findings from cardiac assessments

12-Lead Electrocardiogram (ECG) and Heart Rate Variability Recording (HRV)	• ST-segment abnormalities, • T-wave abnormalities, • Left ventricular hypertrophy, • Left-axis deviation, • Sinus arrhythmia, • Atrial fibrillation/flutter^a^, • Isolated supraventricular/ventricular ectopics, • Bi/trigemini, • Non-sustained ventricular tachycardia or other malignant ventricular arrhythmias^a^, • Bundle branch block, • Prolonged PR interval, • 2^nd^ degree AV block or higher^a^
Echocardiogram	• Valve prosthesis, • Mild valve stenosis, • Moderate to severe valve stenosis^a^, • Mild valvular regurgitation, • Moderate to severe valvular regurgitation^a^, • Impaired LV or RV systolic function^a^, • Severe LV diastolic dysfunction^a^, • Chamber enlargement (e.g., left atrial dilatation, right ventricular dilatation, etc.), • Left or right ventricular hypertrophy LV outflow tract obstruction, • Regional wall motion abnormality^a^, • Intracardiac shunt^a^, • Pericardial effusion^a^, • Pulmonary hypertension, • Mass lesion (vegetation, thrombus, or tumour)^a^, • Myocardial infiltration (suggesting cardiac amyloidosis)^a^, • Presence of congenital heart disease, • Aortic dilation, aortic coarctation^a^, or aortic dissection^a^
Carotid intima-media thickness (cIMT)	• Carotid artery stenosis (≥ 50% diameter), • Carotid atheromatous plaques, • Carotid artery thrombus, • Carotid tumours^a^, • Carotid dissection^a^, • Large thyroid mass (> 1 cm)^a^, • Lymphadenopathy

^a^Indicates a potentially urgent finding, particularly if not previously known about

Duty-of-care guidelines for the DXA scan are also based on previous 2006–2010 reporting used by MRC NSHD [82]. Hip and spine T-scores are analysed and reported to the participant and their GP within two weeks of the scan. The T-scores are categorised as either normal (T > -1.0 SD), osteopenia (-2.5 SD < T ≤ -1.0 SD), or osteoporosis (T ≤ -2.5 SD).

Duty-of-care guidelines for the OCT scan have been established in collaboration with experts at Moorfields Eye Hospital, London [83], and all OCT images are reviewed at Moorfields. A report of any significant incidental findings and advice on the best course of action is provided. Any incidental findings that require immediate action (e.g., tear in periphery, participant symptomatic of floaters) results in an urgent report and contact with the participant and their GP.

## Discussion

The NSHD is the longest continually running British birth cohort. NSHD study participants have been involved in up to 27 data sweeps throughout their lives [10], and, since age 70, a proportion of them have been invited to participate in Insight 46 (this neuroimaging sub-study) and MyoFit 46 (a cardiovascular sub-study) [84]. As such, there are a wealth of data available to investigate genetic, life course, and concurrent factors that may influence brain health and cognition in a person in their 70 s.

The primary objectives of Insight 46 are to identify presymptomatic AD and associated biomarkers, investigate life course and genetic influences on symptom onset and progression, and provide a critical evidence base for future research. Furthermore, we hope that this work will inform preclinical AD therapeutic trial design, from inclusion criteria to outcome measures, as well as risk factors that may be targets for drug development. Analyses of cross-sectional (phase 1) and longitudinal data (phase 2) are ongoing, and our analyses published to date have already added to the scientific understanding of several of the primary objectives of Insight 46 [16–20, 26, 27, 37, 56, 85].

Although there are many strengths to using the NSHD as the source of recruitment for Insight 46 (particularly the nearly identical birthdates of the cohort members and the rich life course dataset), there are also some inherent limitations. First, there is a lack of ethnic diversity, as all singleton births enrolled in the NSHD were white; this was reflective of the British population in 1946 but not the diversity of the current UK population. Second, there is a bias in Insight 46 toward study members who are healthy enough to travel to London, as travel can be particularly difficult for participants in areas further afield such as Scotland and Cornwall. James et al*.* [14] has described some characteristics related to participation rates for phase 1. As the burden of the study visit increases with each phase (phase 1 = one day of assessments, phase 2 = 1.5 days of assessments, phase 3 = two days of assessments) and as the cohort continues to age, we expect to see an increase in the number of participants who are not willing or able to travel to London for a clinic visit.

Moving forward there are plans to further enrich the data collection associated with Insight 46. This will include inviting approximately 250 Insight 46 participants to install an in-home sleep mat to passively collect sleep data for several years. Additional NSHD data collections are also underway, which will feed into the detailed physical and cognitive data collected for Insight 46. These include home visits to participants unable to travel to London and the use of Cognitron’s online cognitive testing platform [86, 87] for participants who have access to a computer or tablet and an internet connection. Data are available to bona fide researchers upon request to the NSHD Data Sharing Committee via a standard application procedure. Further details can be found at http://www.nshd.mrc.ac.uk/data.

## Data Availability

Data generated in Insight 46 will be made available one year after each phase of data collection is completed, allowing sufficient time for quality control, and confidentiality of imaging data to be assured. Data will be made available to bona fide researchers through MRC Unit for Lifelong Health and Ageing’s standard security processes, and according to established NSHD data sharing guidelines (http://www.nshd.mrc.ac.uk/data). Requests for data will be considered by a data sharing committee on the basis of quality, scientific priorities and overlapping interests.

## Abbreviations

Aβ: ß-amyloid
AD: Alzheimer’s disease
ATT: Arterial transit time
AMIPB: Adult Memory and Information Processing Battery
ASL: Arterial spin labelling
BRAIN: Bradykinesia akinesia incoordination
BVP: Blood volume pulse
CBF: Cerebral blood flow
CDR: Clinical Dementia Rating
cIMT: Common carotid artery intima-media thickness
CSF: Cerebrospinal fluid
DSST: Digit-Symbol Substitution Test
DWI: Diffusion-weighted imaging
DXA: Dual-energy X-ray absorptiometry
EDA: Electrodermal activity
EPI: Echo-planar imaging
ESS: Epworth Sleepiness Scale
ECG: Electrocardiogram
FEV1: Forced expiratory volume in one second
FLAIR: Fluid attenuated inversion recovery
FNAME: Face—Name Associative Memory Exam
fNIRS: Functional near-infrared spectroscopy
FVC: Forced vital capacity
GDPR: General Data Protection Regulations
GFAP: Glial fibrillary acidic protein
GHQ: General Health Questionnaire
GNT: Graded Naming Test
GP: General practitioner
HRA REC: Health Research Authority Research Ethics Committee
HRV: Heart rate variability
IBI: Inter-beat interval
IR-IVM: Inversion recovery intravoxel incoherent motion
IVA: Instant vertebral assessment
LP: Lumbar puncture
MMSE: Mini Mental State Examination
MP2RAGE: Magnetization-prepared 2 rapid acquisition gradient echo
MRI: Magnetic resonance imaging
NSHD: National Survey of Health and Development
OCT: Optical coherence tomography
PBMCs: Peripheral blood mononuclear cells
PCSAL: Pseudo-continuous arterial spin labelling
PEEK: Portable Eye Examination Kit
PET: Positron emission tomography
PSQI: Pittsburgh Sleep Quality Index
PTQ: Perseverative Thinking Questionnaire
PWA: Pulse wave analysis
PWV: Pulse wave velocity
QSM: Quantitative susceptibility mapping
RSBDQ: REM Sleep Behaviour Disorder Questionnaire
SFS: Speech filing system
STAI: State-Trait Anxiety Inventory
SWI: Susceptibility weighted imaging
TSH: Thyroid-stimulating hormone
UCL: University College London
UPDRS: Unified Parkinson’s Disease Rating Scale
UPSIT: University of Pennsylvania Smell Identification Test
WAIS-R: Wechsler Adult Intelligence Scale-revised
WASI: Wechsler Abbreviated Scale of Intelligence
WMS: Wechsler Memory Scale
WMS-R: Wechsler Memory Scale-revised
